# CT radiomics-based long-term survival prediction for locally advanced non-small cell lung cancer patients treated with concurrent chemoradiotherapy using features from tumor and tumor organismal environment

**DOI:** 10.1186/s13014-022-02136-w

**Published:** 2022-11-16

**Authors:** Nai-Bin Chen, Mai Xiong, Rui Zhou, Yin Zhou, Bo Qiu, Yi-Feng Luo, Su Zhou, Chu Chu, Qi-Wen Li, Bin Wang, Hai-Hang Jiang, Jin-Yu Guo, Kang-Qiang Peng, Chuan-Miao Xie, Hui Liu

**Affiliations:** 1 Department of Radiation Oncology, State Key Laboratory of Oncology in South China, Collaborative Innovation Center for Cancer Medicine, Sun Yat-sen University Cancer Center, No.651 Dongfeng Road East, 510060 Guangzhou, China; 2 Department of Imaging Diagnosis and Interventional Center, State Key Laboratory of Oncology in South China, Collaborative Innovation Center for Cancer Medicine, Sun Yat-sen University Cancer Center, No.651 Dongfeng Road East, 510060 Guangzhou, Guangdong China; 3 Department of Cardiac Surgery, The First Affiliated Hospital of Sun Yat-sen University, Guangzhou, Guangdong China; 4 Department of Pulmonary and Critical Care Medicine, The First Affiliated Hospital of Sun Yat-sen University, Guangzhou, Guangdong China; 5 Homology Medical Technologies Inc., Ningbo, Zhejiang China; 6 Guangzhou Xinhua University, Guangzhou, Guangdong China

**Keywords:** Locally advanced non-small cell lung cancer, Radiomics, Machine learning, Long-term survival prediction, Tumor organismal environment.

## Abstract

**Background:**

Definitive concurrent chemoradiotherapy (CCRT) is the standard treatment for locally advanced non-small cell lung cancer (LANSCLC) patients, but the treatment response and survival outcomes varied among these patients. We aimed to identify pretreatment computed tomography-based radiomics features extracted from tumor and tumor organismal environment (TOE) for long-term survival prediction in these patients treated with CCRT.

**Methods:**

A total of 298 eligible patients were randomly assigned into the training cohort and validation cohort with a ratio 2:1. An integrated feature selection and model training approach using support vector machine combined with genetic algorithm was performed to predict 3-year overall survival (OS). Patients were stratified into the high-risk and low-risk group based on the predicted survival status. Pulmonary function test and blood gas analysis indicators were associated with radiomic features. Dynamic changes of peripheral blood lymphocytes counts before and after CCRT had been documented.

**Results:**

Nine features including 5 tumor-related features and 4 pulmonary features were selected in the predictive model. The areas under the receiver operating characteristic curve for the training and validation cohort were 0.965 and 0.869, and were reduced by 0.179 and 0.223 when all pulmonary features were excluded. Based on radiomics-derived stratification, the low-risk group yielded better 3-year OS (68.4% vs. 3.3%, p < 0.001) than the high-risk group. Patients in the low-risk group had better baseline FEV1/FVC% (96.3% vs. 85.9%, p = 0.046), less Grade ≥ 3 lymphopenia during CCRT (63.2% vs. 83.3%, p = 0.031), better recovery of lymphopenia from CCRT (71.4% vs. 27.8%, p < 0.001), lower incidence of Grade ≥ 2 radiation-induced pneumonitis (31.6% vs. 53.3%, p = 0.040), superior tumor remission (84.2% vs. 66.7%, p = 0.003).

**Conclusion:**

Pretreatment radiomics features from tumor and TOE could boost the long-term survival forecast accuracy in LANSCLC patients, and the predictive results could be utilized as an effective indicator for survival risk stratification. Low-risk patients might benefit more from radical CCRT and further adjuvant immunotherapy.

**Trial registration:**

retrospectively registered.

**Supplementary Information:**

The online version contains supplementary material available at 10.1186/s13014-022-02136-w.

## Introduction

Definitive concurrent chemoradiotherapy (CCRT) is the standard treatment for patients with unresectable locally advanced non-small cell lung cancer (LANSCLC). In the past two decades, concomitant regimens achieved promising tumor local control and long-term survival. With improved outcome, the maintenance of an adequate pulmonary function is essential to ensure acceptable quality of life and adjuvant immunotherapy. However, many patients with LANSCLC are diagnosed with pre-existing lung comorbidities, which significantly increases the risk for radiation-induced lung toxicity (RILT) [1, 2].

Most existing RILT prediction models largely focused on clinical prognostic factors (CPFs) and dose-volume histogram parameters [3–5], but remained insufficient. Recently, machine learning methods have been reported to improve the capacity of the predictive modelling [6–9], compared with logistic regression widely used in normal tissue complication probability model.

Moreover, radiomics analysis, attempting to identify computational biomarkers potentially hidden within high-throughput imaging data [10, 11], has been demonstrated the added predictive value for overall survival (OS) [12–14] or RILT [8, 9]. However, most of them rely on the radiomic information from tumor or its surrounding peritumoral region, few studies have been designed based on the radiomics analysis of tumor organismal environment (TOE).

Similar to other published reports [15, 16], our previous study [17] indicated that pulmonary function test (PFT) was significantly related to patients’ long-term survival. However, it failed to predict progression-free survival (PFS). Even though patients with worse FEV1/FVC% or DLCO% showed a high objective response rate (ORR) to CCRT, their survival outcomes were still poor, hinting that TOE, the status of lungs in the case of LANSCLC might play an indispensable role in the prognostic prediction after CCRT. As some patients could not tolerate well with PFT, radiomics analysis using machine learning method might be an effective technique to investigate the relationship of tumor and TOE, due to its accessibility.

In this study, we utilized computed tomography (CT) images before CCRT to develop an image-based machine learning framework to analyze the relationship of primary lung tumor and bilateral lungs for long-term survival prediction in LANSCLC. To balance the training accuracy and predictive capability using relative small number of patient samples, an integrated feature selection and model training (IFSMT) approach was developed to extract the most critical quantitative radiomic features from both tumor and lungs. A radiomic-based risk stratification was built to distinguish high-risk and low-risk patients and provided evidence for clinical decision making.

## Methods

### Study population

Consecutive patients irradiated for lung cancer from September 2011 to April 2019 in our institution were retrospectively screened. Inclusion criteria included: (1) histologically confirmed NSCLC; (2) unresectable stage III disease (AJCC/UICC 8th staging criteria) proven by chest and upper abdominal CT, brain magnetic resonance imaging (MRI), bone scan and/or positron emission tomography-computed tomography (PET-CT); (3) definitive radiotherapy with concurrent chemotherapy was administered; (4) stay followed-up no less than 6 months since the start of radiotherapy (unless death or disease progression was documented); (5) complete clinical records. Patients that met the inclusion criteria were randomly assigned into the training and validation cohort, with the numbers at a ratio 2:1.

### Planning CT image acquisition

The four-dimensional (4D) planning CT scan was performed 1–2 weeks prior to treatment, using multiple CT simulation positioning machines with varied parameter settings in our institution (detailed in **Additional File 1**). Ten phases of the breathing cycle were reconstructed, including: 0%, 10%, 20%, 30%, 40%, 50%, 60%, 70%, 80%, and 90%. The segmentation and radiomics were then performed on the 20% phase (middle exhale phase) with a consistent mediastinum/lung window level setting.

### Radiotherapy and concurrent chemotherapy

Patients were positioned supine and immobilized in a vacuum pad. They were scanned from the Atlas to the second lumbar vertebra level with 0.3-0.5 cm thickness slices to obtain the stimulation CT images. The respiration motion was recorded by performing 4DCT scanning. The maximum intensity projection images were reconstructed using the images collected in 10 phases of respiratory cycle. Gross tumor volume (GTV) was delineated to cover the tumor and involved regional nodes visible on each phase of the 4DCT. The total volumes of GTVs across the 10 respiratory phases CT composed the internal target volume (ITV). Planning target volumes (PTVs) were created by expanding GTV and clinical target volume with 6 mm. Lungs were delineated according to the atlases for organs at risk (OARs) in thoracic radiation therapy [18], but GTV was excluded from the lung delineation. A dose of 60-76 Gy was prescribed to PTV-GTV in 22–33 fractions, with 2-3 Gy per fraction performed once daily, using intensity modulated radiation therapy technique. The dose constraints for OARs were: V20 < 35% for lungs; mean lung dose < 19 Gy; maximun dose (Dmax) of esophagus < 105% prescription dose; Dmax of spinal cord < 46 Gy; V30 < 40% for heart.

All patients received platinum-based double agents weekly or every three weeks. The regimens included docetaxel/paclitaxel/etopside/pemetrexed plus platinum.

### Evaluation and follow-up

The baseline characteristics of each patient before entry were reviewed attentively and extracted from their medical records, including blood tests, PFT, blood gas analysis (BGA) and radiologic tests. All included patients received regular radiologic follow-up, including chest and upper abdominal CT and brain MRI performed every 3 ~ 6 months in the first 2 years, and every 6 ~ 12 months thereafter. PET-CT, bone scan, and biopsy were recommended if clinically required. The responses to CCRT were first assessed by an independent radiation oncologist and confirmed by a senior physician at 4 ~ 6 weeks post CCRT, based on Response Evaluation Criteria in Solid Tumors 1.1. Another senior radiologist was consulted for disagreement. Therapeutic toxicities were graded and recorded according to Common Terminology Criteria for Adverse Events 4.0.

### OS modelling procedures

**Fig. 1:**
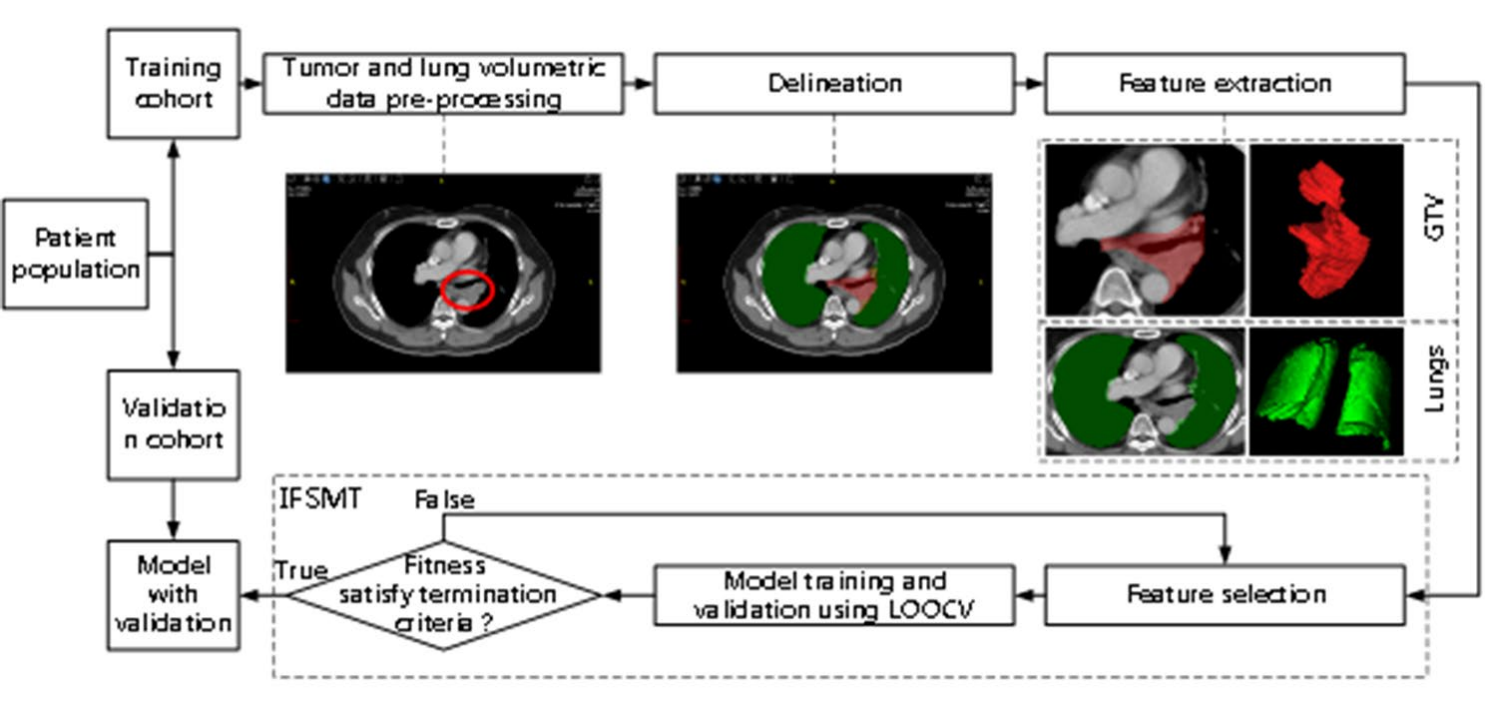
Schematic overview of the integrated feature selection and model training (IFSMT) approach. IFSMT approach consisted of five steps: (1) volumetric data pre-processing; (2) delineation; (3) feature extraction; (4) integrated feature selection and model training; (5) model validation using leave-one-out cross-validation (LOOCV).

The whole procedures were illustrated in Fig. 1. For both cohorts of patients, the regions of interest (ROIs) corresponding to GTV and lungs were delineated by an auto-contouring software tool CezanneDraw™ v1.0 (Homology Medical, Ningbo, China, 2020) using the CT slices and manually modified by radiation oncologists if necessary. One 3D bounding box was fitted for each ROI. And inside the bounding box, the CT values of the ROI voxels were retained while the values of other voxels were marked by zero. CT values of voxels in each bounding box were then interpolated to a resolution of 1 mm×1 mm×5 mm and resampled into 400 discrete values (called bins) with absolute discretization from − 1000 to 3000 Hounsfield units, leading to a fixed bin size of 10 Hounsfield units.

A total of 92 tumor-related and lung-related features were then computed for both ROIs and used as the input feature pool for the machine learning framework by the LIFEx software (version 3.44) [19]. The imaging-based features covered two categories of texture features and first order features. The texture features consisted of four sub-categories of matrix based texture features. These matrices included the grey-level co-occurrence matrix (GLCM), neighborhood grey-level different matrix (NGLDM), grey-level run length matrix (GLRLM) and grey-level zone length matrix (GLZLM). The first order features included indices from shape, indices from histogram and conventional indices.

**Fig. 2:**
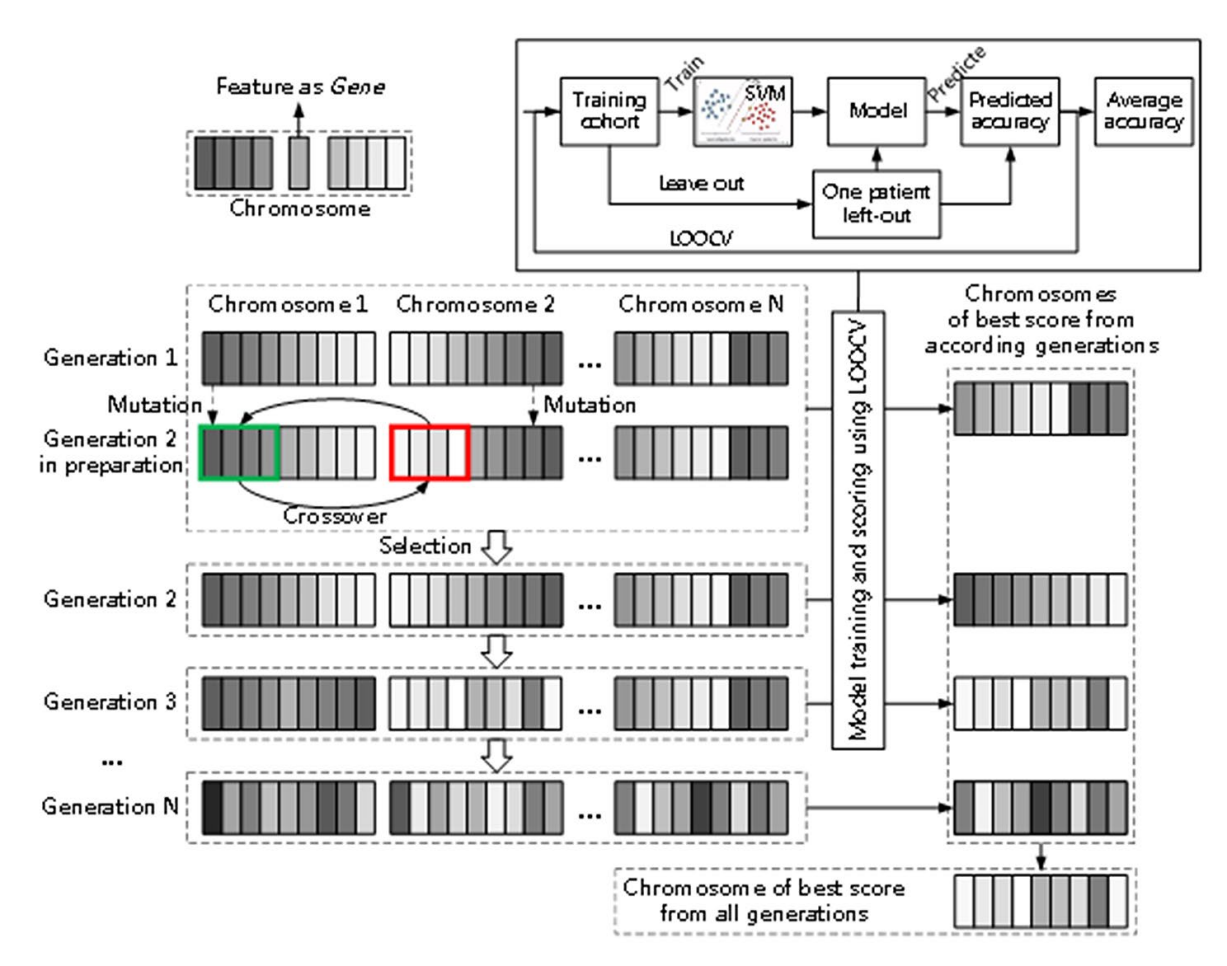
Schematic overview of the genetic algorithm (GA) in the integrated feature selection and model training (IFSMT) approach. A chromosome is scored with LOOCV-SVM. The chromosomes of higher scores may go through mutation and crossover to make new ones to replace those of lower scores. Collect the chromosome of best score from each generation into a group. And the best one in the group is the result of the model. Abbreviation: LOOCV, leave-one-out cross-validation; SVM, support vector machine

The machine learning based classification method used to predict the two-class 3-year survival status for each individual patient was support vector machine (SVM) [20]. The SVM mapped the features of training data into a high-dimensional feature space through a kernel function and utilizes a hyper-plane to optimally separate the training data points into two categories. To reduce the possibility of overfitting, only a subset of features from the feature pool could be selected for the input of SVM. In this study, the IFSMT approach was developed to maximize the fitting accuracy and minimize the overfitting potential. This posteriori approach applied the genetic algorithm (GA) for the feature selection, which was illustrated in Fig. 2 and Additional File 2. A chromosome represents a feature template working with SVM of certain configuration for diagnosing purpose. The SVM is implemented in leave-one-out cross-validation (LOOCV) fashion to score a chromosome. In each generation, the chromosomes of higher scores may go through mutation, partially changing feature encoding, and crossover, partially exchanging feature encoding, to make new ones to replace those of lower scores. Collect the chromosome of best score from each generation into a group. And the best one in the group is the result of the model. Manual reconfiguration of SVM is not included in the model.

Once the optimal set of features was determined, the SVM models were trained again on the training cohort. In this study, after extensive experimental comparisons, the linear kernel was chosen for SVM and optimal hyper parameters of the SVM (C, ε and γ) were determined through exhaustive search in the parametric space. Receiver operating characteristics (ROC) curves were obtained by varying threshold of the decision variable, the signed distance to decision hyper-plane. Area under curve (AUC) for each ROC was calculated for training cohort. The trained models were then used to predict the survival status for each individual patient in the validation cohort, and ROCs and their corresponding AUCs were also calculated. All the above feature selection and machine learning approaches were implemented on the cloud-based clinical data service platform iRAAS^®^ v2.0 (Homology Medical, Ningbo, China, 2020).

To assess the importance of each selected feature to the accurate prediction of the clinical outcome, a one-by-one feature evaluation procedure was designed. This procedure tested the importance of each feature by deleting each feature from the selected feature set and calculating the reduction of the AUC for the model trained with the original selected features except this specific feature. This reduction of model performance was used as the importance weight (IW) of this feature. All the selected features were then sorted according to their IWs. To further assess the importance organismal features, the AUC for the model trained with the original selected features excluding all the lung-related features were also calculated.

### Statistical methods

OS was defined as the time from radiotherapy start to the last follow up, which ended at November 30th, 2021, or death. A t-test was used to determine if there was significant difference between the means of continuous variables, while Fisher’s exact test was performed to reveal the difference in distribution between two groups of categories variables. The association between radiomic features and PFT/BGA indicators was examined using Pearson’s correlation coefficient. A p-value < 0.05 (two-sided) were considered as statistically significant. Missing data were excluded from the statistical analysis. Statistics were performed using SPSS 22.0 (IBM, Chicago, IL, USA).

To report the model fitting accuracy and the prediction capability, the true positive rate (TPR), true negative rate (TNR), F1 score, overall prediction accuracy, average prediction accuracy for the training cohort and validation cohort were calculated based on the SVM model. Herein, death is marked as the positive. The overall prediction accuracy was expressed as the number correctly predicted patients / the number of all patients; and the average prediction accuracy = (TPR + TNR)/2.

To assess the prognostic value of the survival status model, the predicted 3-year survival status was adopted respectively as the clinical risk estimator to stratify the patients into the high-risk and low-risk groups. Patients with negative predicted survival status were classified into the low-risk group and the others with positive predicted survival status into the high-risk group. Kaplan-Meier curves for both groups were displayed to illustrate its effectiveness and log-rank test was performed.

## Results

### Patient characteristics

A total of 298 LANSCLC patients were included for analysis, with 200 in the training cohort and 98 in the validation cohort. The baseline and treatment-related characteristics were comparable between these two cohorts (Additional File 3). There were 57 females and 241 males in the whole cohort, with the median age of 59 years (range, 28–81 years). Squamous cell carcinoma was the predominant histologic type both in the training (46.5%) and validation (62.2%) cohorts.

### OS modelling

With the median follow-up of 27.7 (range, 4.0 ~ 122.7) months for all and 67.0 months (range, 36.2 ~ 122.7 months) for event-free patients, our cohort demonstrated the estimated median OS of 27.6 (95% confidence interval (CI), 22.3 ~ 33.0) months, and the 3-year OS rate was 43.0% (95%CI, 37.3%~48.7%).

As shown in Table 1, the overall prediction accuracy for 3-year survival status was 92.50% and 85.71%, and the AUC of the ROC was 0.965 and 0.869, respectively, in the training and validation cohort.

**Table 1:** OS Training and validation accuracy using all selected features or without pulmonary features

OS status accuracy	Training cohort		Validation cohort	
	**All features**	**Without pulmonary features**	**All features**	**Without pulmonary features**
AUC	0.965	0.786	0.869	0.646
TNR (%)	95.00	74.00	92.86	75.00
TPR (%)	90.00	68.00	82.86	50.00
F1 score	0.923	0.701	0.892	0.625
Average accuracy (%)	92.50	71.00	87.86	62.50
Overall accuracy (%)	92.50	71.00	85.71	57.14

Abbreviations: OS, overall survival; AUC, area under curve; TNR, true negative rate; TPR, true positive rate

### Stratification of patients in the validation cohort with machine learning model

**Fig. 3:**
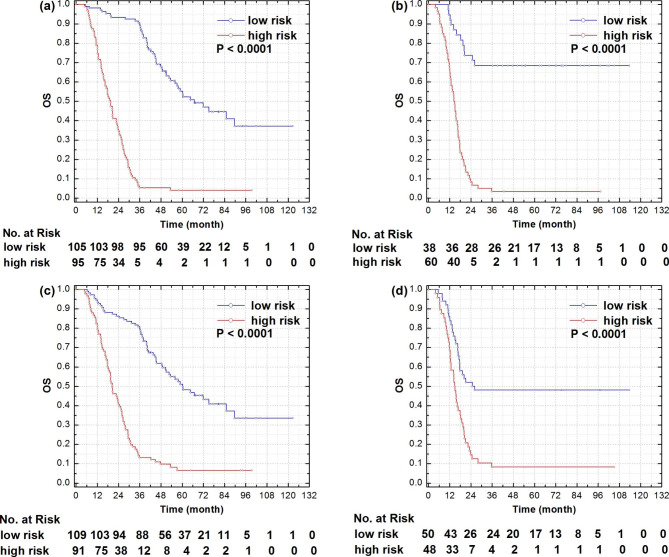
Kaplan-Meier curves for the training and validation cohort, with all selected features (**a**, **b**), and without pulmonary features (**c**, **d**), respectively

**Fig. 4:**
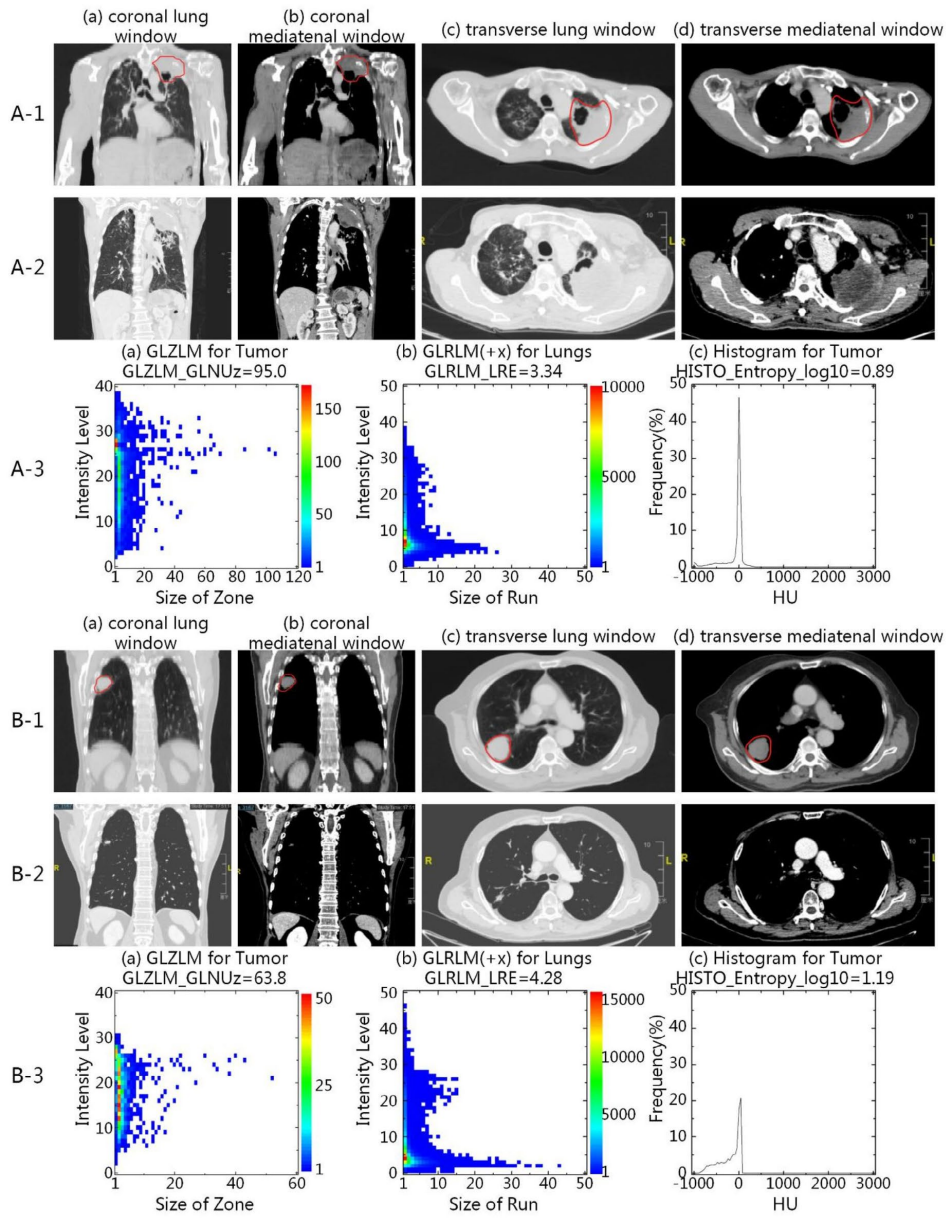
Two patients from the high-risk (**A**) and low-risk group (**B**). From the series CT images, there were discernible distinction observed in tumor and pulmonary status between the two cases. High-risk patient had heterogeneous primary lung tumor and chronic obstructive pneumonia (A-1), while low-risk patient had relatively homogeneous primary lung tumor and better pulmonary condition (B-1). Significant tumor remission was achieved in low-risk patient without obvious radiation pneumonitis after CCRT (B-2), while high-risk patient had stable disease and developed Grade 2 radiation pneumonitis in bilateral lungs (A-2). The GLZLM matrices for tumor, GLRLM matrices along + x axis for lungs, and histograms of HU values for tumor (A-3, B-3) were displayed. It was found that the short homogeneous runs and the non-uniformity of the grey-levels (CT value) were emphasized for high-risk patient compared to those of low-risk patient

In the validation cohort, 60 (61.2%) of 98 patients were stratified into the high-risk group and 38 (44.1%) into the low-risk group. CCRT was more successful in patients in the low-risk group than those in the high-risk group. The ORR was 84.2% (32/38) and 66.7% (40/60) in the low-risk and high-risk group, respectively (p = 0.003) (Additional File 4). And the low-risk group yielded better 3-year OS (68.4% versus 3.3%, p < 0.001, log-rank) than the high-risk group (Fig. 3B). What’s more, the rate of Grade ≥ 2 pneumonitis was 31.6% (12/38), versus 53.3% (32/60) (p = 0.040) in the low-risk and high-risk group. The typical presentation of two patients in the low-risk and high-risk group was illustrated in Fig. 4.

### Correlation of selected radiomic features to the model performance

**Fig. 5:**
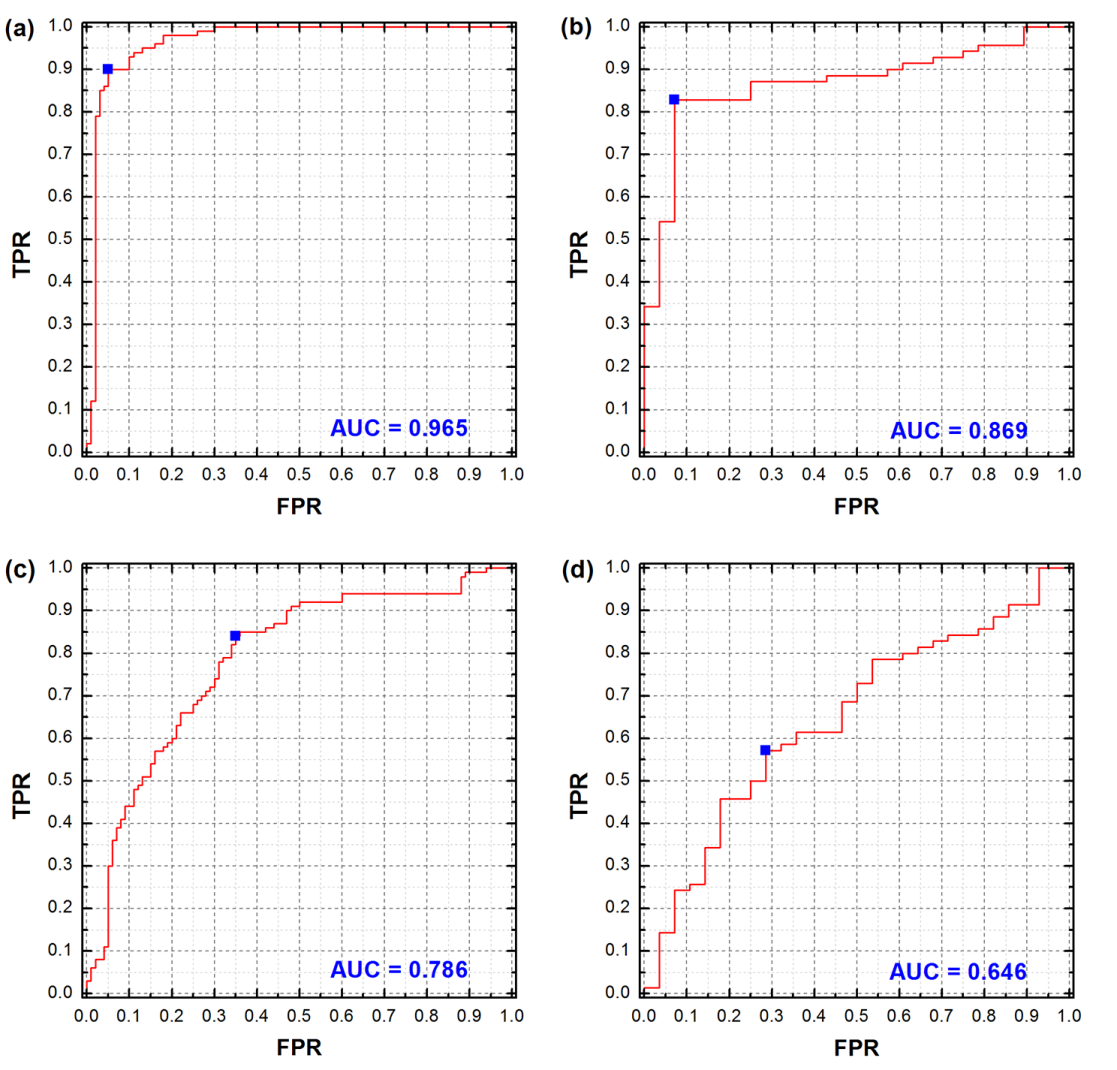
The receiver operating characteristics (ROC) curves for the training and validation cohort, with all selected features (**a**, **b**), and without pulmonary features (**c**, **d**), respectively

A total of 9 features were selected in the proposed model, including 5 tumor-related features and 4 lung-related features. In Table 2, the IW of each selected feature for both training and validation cohorts were listed in the order from high to low. The imaging features from lungs ranked at 2nd, 4th, 5th and 8th in the all 9 features in the training cohort, and 1st, 3rd, 6th, and 8th in the validation cohort. When all pulmonary features were excluded from the selected feature set, the AUCs for the training and validation cohorts were reduced by 0.179 and 0.223, respectively (Fig. 5). Figure 4 showed two patients in the low-risk and high-risk groups.

**Table 2:** Selected features and their importance rank for the training and validation cohort

Importance rank	Training cohort			Validation cohort		
	**Selected features**	**Category**	**IW**	**Selected features**	**Category**	**IW**
1	GLRLM_SRE	Tumor	0.0451	SHAPE_Volume_mL	Lung	0.1439
2	SHAPE_Volume_mL	Lung	0.0426	GLZLM_GLNUz	Tumor	0.1122
3	CONV_SUVstd	Tumor	0.0177	GLRLM_RP	Lung	0.0776
4	CONV_SUVstd	Lung	0.0169	GLRLM_SRE	Tumor	0.0643
5	GLRLM_RP	Lung	0.0143	HISTO_Entropy_log10	Tumor	0.0541
6	GLZLM_GLNUz	Tumor	0.0126	CONV_SUVstd	Lung	0.0372
7	HISTO_Entropy_log10	Tumor	0.0121	TLG_mL	Tumor	0.0224
8	GLRLM_LRE	Lung	0.0036	GLRLM_LRE	Lung	0.0066
9	TLG_mL	Tumor	0.0006	CONV_SUVstd	Tumor	＜0.0001

Abbreviations: IW, importance weight; GLRLM, grey-level run length matrix; SRE, short-run emphasis; RP, run percentage; GLZLM, grey-level zone length matrix; GLNUz, gray-level non-uniformity for zone; LRE, long-run emphasis; TLG, total lesion glycolysis

### Correlation of radiomic features to the PFT/BGA indicators

**Fig. 6:**
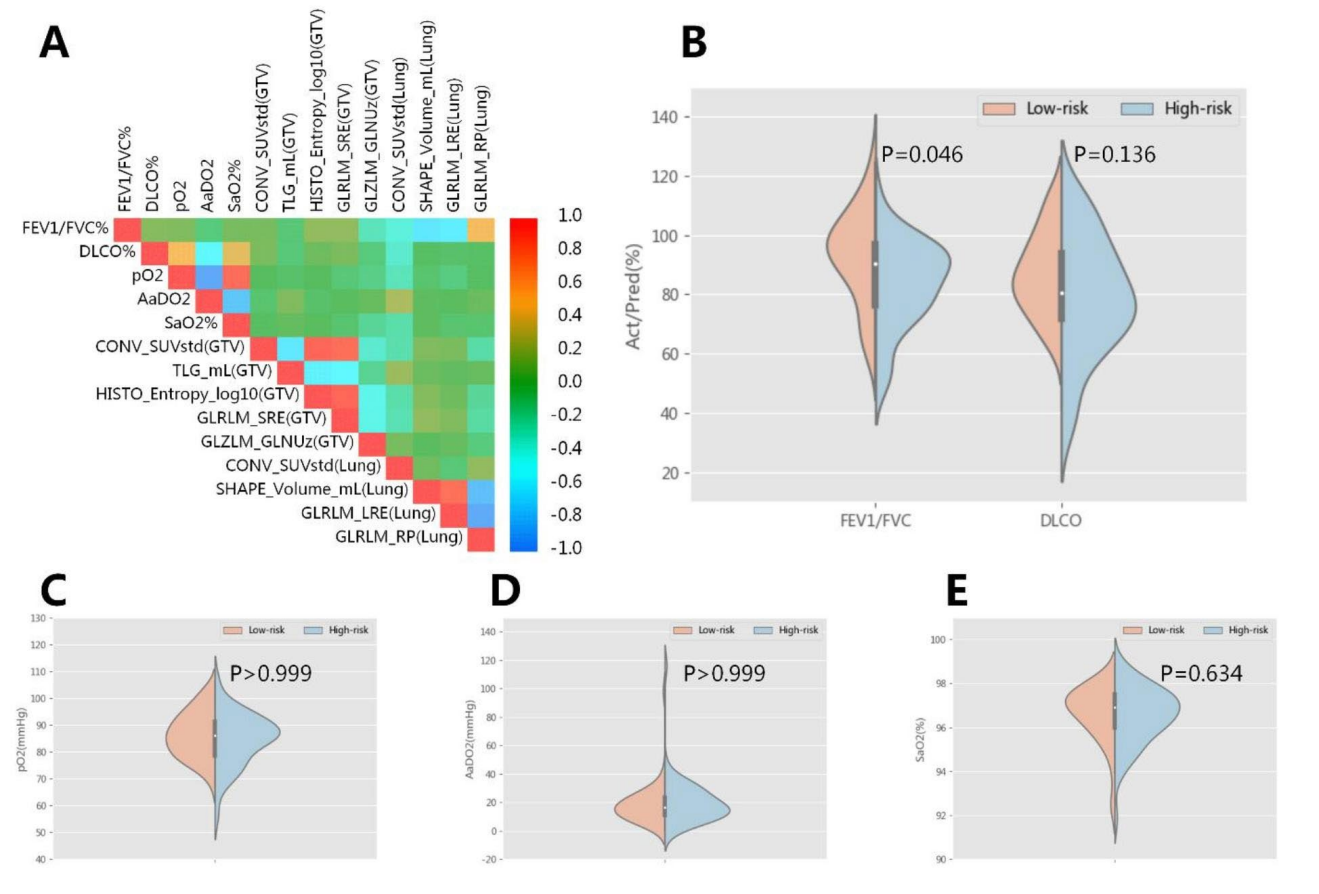
Pearson correlation coefficient heatmap for radiomic features and PFT/BGA indicators (**A**), and the distribution of PFT/BGA indicators between the low-risk and high-risk group (**B**-**E**). A. The FEV1/FVC% had modest correlation with three pulmonary features (SHAPE_Volume_mL, GLRLM_LRE and GLRLM_RP) (all Pearson correlation >|0.45|), and mild correlation with CONV_SUVstd of lungs and GLZLM_GLNUz of tumor (all Pearson correlation >|0.25|). Other PFT/BGA indicators and radiomic features were not well correlated. B-E. Patients in the low-risk group had better baseline FEV1/FVC% (median, 96.3% vs. 85.9%, p = 0.046) compared with those in the high-risk group. No significant difference of DLCO% (median, 84.3% vs. 77.5%, p = 0.136), pO2 (median, 86.0 vs. 86.0 mmHg, p > 0.999), AaDO2 (median, 18.0 vs. 18.0 mmHg, p > 0.999) and SaO2 (median, 96.8% vs. 96.5%, p = 0.634) was found. Abbreviation: PFT, pulmonary function test; BGA, blood gas analysis

Pearson correlation analysis (Fig. 6A) demonstrated that FEV1/FVC% had modest correlation with three pulmonary features (SHAPE_Volume_mL, GLRLM_LRE and GLRLM_RP) (all Pearson correlation >|0.45|), and mild correlation with CONV_SUVstd of lungs and GLZLM_GLNUz of tumor (all Pearson correlation >|0.25|). Other PFT/BGA indicators and radiomic features were not well correlated.

Patients in the low-risk group had better baseline FEV1/FVC% (median, 96.3% vs. 85.9%, p = 0.046) compared with those in the high-risk group (Fig. 6B). Kaplan-Meier analysis indicated that better baseline FEV1/FVC% (p = 0.006) and SaO2 (p = 0.039) could exhibit superior OS, DLCO% (p = 0.063) had a tendency to be associated with OS, however, pO2 (p = 0.110) and AaDO2 (p = 0.299) failed to predict OS (Additional File 5).

### Dynamic changes of lymphocyte counts before and after CCRT

Although there was no significant difference in lymphocyte counts before CCRT (median, 1650 vs. 1650 cells/mm^3^, p > 0.99) between the low-risk and high-risk group (Additional File 6), patients in the low-risk group had less Grade ≥ 3 lymphopenia (63.2% vs. 83.3%, p = 0.031) during CCRT, and more patients in the low-risk group could recover to normal level (≥ 1000 cells/mm^3^) at 4 ~ 6 weeks post CCRT (71.4% vs. 27.8%, p < 0.001). Kaplan–Meier curves demonstrated that better recovery to normal level (≥ 1000 cells/mm^3^) at 4 ~ 6 weeks post CCRT (3-year OS rate, 47.5% versus 14.3%, p = 0.001) was a prognostic factor of OS (Additional File 5).

## Discussions

Application of radiomics to the long-term survival prediction for LANSCLC after CCRT is a reasonable extension under the background of the field-wide adoption of machine learning methods. Other than previous works focused on the features from tumor and peritumoral tissue, the relationship between tumor and TOE is increasingly attached importance. Significant association was found between pulmonary function and radiomic features extracted from the lungs of CT images [21–23]. In current study, the long-term survival forecast accuracy of LANCLC patients after CCRT was demonstrated to be boosted by integrating primary tumor characteristics and pulmonary features from pretreatment CT images. Based on the CT-based predictive model, patients could be precisely stratified into the low-risk and high-risk group before treatment, which should be considered in individualized treatment decision-making process.

From the importance rank of the selected features, it could be confirmed that two features from tumor, GLRLM_SRE and GLZLM_GLNUz which represent the inhomogeneity of CT images [19], remained important factors determining OS, which were consistent with published literatures [24, 25]. Meanwhile, the ranking of pulmonary features underlined their indispensable role in the OS forecast. Our results of the significant difference between fitting and prediction accuracies with and without pulmonary features in model performance further support this finding, implying that the TOE, herein the pulmonary environment, might have a significant impact in LANSCLC patients with large tumor burden and limited pulmonary function. Accordingly, the relatively longer OS for patients with healthier pulmonary status could possibly contribute to their more tolerance to radical CCRT and less incidence of severe lung toxicities.

PFT have been reported to predict the risk of RILT after CCRT [26–29]. Our previous work showed that FEV1/FVC% and DLCO% were prognostic factors for long-term survival but not for PFS [17], implying that long-term survival outcomes might not be achievable due to detriment of pulmonary function even though patients had good early response to CCRT. To further interpret the underlying role of these selected radiomic lung features, the correlation between radiomic features and PFT/BGA indicators were explored in depth and it was confirmed that FEV1/FVC% was well correlated with radiomic pulmonary features. This correlation between the pulmonary ventilation function and selected radiomic pulmonary features for OS prediction reaffirms the findings in Occhipinti et al.’s study that the changes in lung function, such as bronchial thickening and honeycombing, can be mechanistically explained based on morphological CT features [23]. And it might additionally imply that the tumor not only interacts with cells in its immediate vicinity, but also communicates with the entire host organ [30], just as suggested by a prior study [31] that the tumor and TOE could possibly interact in a bi-directional way.

In the aspect of methodology, the machine learning framework in this study used SVM combined with the proposed IFSMT approach to iteratively select features using GA and improve the accuracy of the prediction model. Our avoidance of topical deep-learning frameworks, such as deep convolutional neural network, is due to the intrinsic weaknesses of overfitting and blackbox for these frameworks. To ease the problem of overfitting, the deep-learning frameworks are more suitable for the learning tasks armed with big data as learning samples. However, the number of patients in current study for model training was relatively small, which intensively restricts the application of deep-learning frameworks which may have millions of parameters and thousands of decision making variables. The SVM is equivalent to an optimized three-layer neural network with only one hidden layer. This simplified neural network architecture substantially reduces the potential of overfitting. Additionally, in contrast with the problem of blackbox for deep learning framework, the features used in modeling are explicitly created and selected with the IFSMT approach. Therefore, each feature had an explicit clinical or physical meaning relevant to image of a specific ROI, which made it easy to apprehend the behind-the-scene mechanism of the survival status prediction and directly related the comprehensible clinical and image oriented indices to the clinical outcome. The effectiveness of IFSMT approach had been demonstrated by high AUC values achieved for the survival status prediction.

The most recent work on prognostic model for the survival outcome for NSCLC patients treated with CCRT demonstrated that pretreatment CT texture features provided prognostic information beyond CPFs [12]. However, it didn’t provide the result in terms of AUC or employ the validation cohort. In another predictive model conducted by Dehing-Oberije C et al. [32], which used CPF indices only, the AUC was 0.74 for the training cohort, 0.75 and 0.76 for the two separate validation cohorts. The improvement of model performance by imaging features in current study is discernible with the AUCs of 0.965 and 0.869 for the training and validation cohort, which could be attributed to inclusion of the image-based pulmonary features.

What’s more, the predictive OS results using imaging features in our study with machine learning could be utilized as an effective indicator for the survival risk stratification of these patients, which could potentially individualize CCRT regimen and adjuvant treatment from the perspective of personalized medicine. For example, immunotherapy has evolved into a standard adjuvant treatment option for LANSCLC patients treated with definitive CCRT. Based on the promising results of the phase III PACIFIC study [33, 34], adjuvant immunotherapy resulted in a significant prolonged PFS and OS for those patients. To be noticed, the most common grade 3 or 4 adverse event in the durvalumab arm was pneumonia (4.4%), followed by pneumonitis or radiation pneumonitis (3.4%), and Asian patients seemed to have a higher rate of any grade pneumonitis (73.6%) and severe pneumonitis (5.6%) [35]. Thus, based on the survival risk stratification of LANSCLC patients in this study, low-risk patients might have several potential advantages for adjuvant immunotherapy: (1) supporting role of better pulmonary function and quality of life; (2) superior tumor remission with less pulmonary toxicities; (3) less severe lymphopenia during CCRT and better recovery of lymphopenia from CCRT. However, for high-risk LANSCLC patients who had worse baseline FEV1/FVC%, higher rate of Grade ≥ 3 lymphopenia during CCRT, worse recovery of lymphopenia from CCRT, and higher incidence of radiation-induced pneumonitis, radical CCRT or further adjuvant immunotherapy might not be feasible because of poor organ functions and high probability of severe complications. Therefore, pretreatment radiomics-based risk stratification of LANSCLC patients using features from tumor and TOE could provide direct evidences to effectively support the treatment decision making.

It should also be noted that there were a few limitations in this study. First, the absence of external validation was the major disadvantage. Nevertheless, multiple CT simulation machines were available in our institution (Additional File 1). The high AUC values were generated from these different scanners with varied parameter settings, demonstrating the great robustness of our model. Besides, Zhao et al. considered that radiomic features in lung cancer were reproducible over a wide range of imaging settings [36]. Multicenter validations with larger samples are warranted for the ultimate application of this model clinically. Second, there might be some variability in multiple observer delineations in our study. E et al. reported that although the ROIs delineation tended to be different between individual experts, an overall high AUC value could still be achieved [37]. Third, we focused only on the radiomic analysis of pretreatment planning CT in this study, and other imaging modalities, such as PET-CT [38] and MRI, still need to be investigated as to whether they could also yield complementary information which would facilitate more accurate predictive models.

## Conclusion

Pretreatment CT-based radiomics features from tumor and TOE could improve the long-term survival forecast accuracy in LANSCLC patients treated with CCRT using machine learning. The predictive results could be utilized as an effective indicator for the stratification of these patients into the low-risk and high-risk groups. It was further confirmed that patients in the low-risk group had better baseline FEV1/FVC%, less severe lymphopenia during CCRT, better recovery of lymphopenia from CCRT, lower incidence of radiation-induced pneumonitis, superior tumor remission and long-term survival, which might suggest more benefit for these patients from radical CCRT or further adjuvant immunotherapy.

## Electronic supplementary material

Below is the link to the electronic supplementary material.

Additional File 1. 4DCT image acquisition

Additional File 2. Genetic algorithm

Additional File 3 Patient characteristics

Additional File 4. Tumor response after CCRT in the low-risk and high-risk group

Additional File 5.

Additional File 6.

## Data Availability

The datasets used and analysed during the current study are available from the corresponding author on reasonable request.

## Abbreviations

CI: confidence interval
CCRT: concurrent chemoradiotherapy
LANSCLC: locally advanced non-small cell lung cancer
RILT: radiation-induced lung toxicity
CPF: clinical prognostic factor OS overall survival
TOE: tumor organismal environment
PFT: pulmonary function test
PFS: progression-free survival
ORR: objective response rate
CT: computed tomography IFSMT integrated feature selection and model training
MRI: magnetic resonance imaging
PET-CT: positron emission tomography-computed tomography
4D: four-dimensional
GTV: gross tumor volume
ITV: internal target volume
PTV: planning target volume
OAR: organ at risk
BGA: blood gas analysis
ROI: regions of interest
GLCM: grey-level co-occurrence matrix
NGLDM: neighborhood grey-level different matrix
GLRLM: grey-level run length matrix
GLZLM: grey-level zone length matrix
SVM: support vector machine
GA: genetic algorithm
LOOCV: leave-one-out cross-validation
ROC: receiver operating characteristics
AUC: area under curve
IW: importance weight TPR true positive rate
TNR: true negative rate

## References

[1] Zhou Z, Song X, Wu A, Liu H, Wu H, Wu Q (2017). Pulmonary emphysema is a risk factor for radiation pneumonitis in NSCLC patients with squamous cell carcinoma after thoracic radiation therapy. Sci Rep.

[2] Li F, Zhou Z, Wu A, Cai Y, Wu H, Chen M (2018). Preexisting radiological interstitial lung abnormalities are a risk factor for severe radiation pneumonitis in patients with small-cell lung cancer after thoracic radiation therapy. Radiat Oncol.

[3] Roach III M, Gandara DR, Yuo HS, Swift PS, Kroll S, Shrieve DC (1995). Radiation pneumonitis following combined modality therapy for lung cancer: analysis of prognostic factors. J Clin Oncol.

[4] Bradley JD, Hope A, El Naqa I, Apte A, Lindsay PE, Bosch W (2007). A nomogram to predict radiation pneumonitis, derived from a combined analysis of RTOG 9311 and institutional data. Int J Radiat Oncol Biol Phys.

[5] Hope AJ, Lindsay PE, El Naqa I, Alaly JR, Vicic M, Bradley JD (2006). Modeling radiation pneumonitis risk with clinical, dosimetric, and spatial parameters. Int J Radiat Oncol Biol Phys.

[6] Valdes G, Solberg TD, Heskel M, Ungar L, Simone II CB (2016). Using machine learning to predict radiation pneumonitis in patients with stage I non-small cell lung cancer treated with stereotactic body radiation therapy. Phys Med Biol.

[7] Yakar M, Etiz D, Metintas M, Ak G, Celik O (2021). Prediction of Radiation Pneumonitis With Machine Learning in Stage III Lung Cancer: A Pilot Study. Technol Cancer Res Treat.

[8] Bourbonne V, Da-Ano R, Jaouen V, Lucia F, Dissaux G, Bert J (2021). Radiomics analysis of 3D dose distributions to predict toxicity of radiotherapy for lung cancer. Radiother Oncol.

[9] Puttanawarut C, Sirirutbunkajorn N, Tawong N, Jiarpinitnun C, Khachonkham S, Pattaranutaporn P (2022). Radiomic and Dosiomic Features for the Prediction of Radiation Pneumonitis Across Esophageal Cancer and Lung Cancer. Front Oncol.

[10] Lee G, Lee HY, Park H, Schiebler ML, van Beek EJR, Ohno Y (2017). Radiomics and its emerging role in lung cancer research, imaging biomarkers and clinical management: State of the art. Eur J Radiol.

[11] Thawani R, McLane M, Beig N, Ghose S, Prasanna P, Velcheti V (2018). Radiomics and radiogenomics in lung cancer: A review for the clinician. Lung cancer (Amsterdam Netherlands).

[12] Fried DV, Tucker SL, Zhou S, Liao Z, Mawlawi O, Ibbott G (2014). Prognostic value and reproducibility of pretreatment CT texture features in stage III non-small cell lung cancer. Int J Radiat Oncol Biol Phys.

[13] Sun W, Jiang M, Dang J, Chang P, Yin FF (2018). Effect of machine learning methods on predicting NSCLC overall survival time based on Radiomics analysis. Radiation Oncol (London England).

[14] Lu MT, Ivanov A, Mayrhofer T, Hosny A, Aerts H, Hoffmann U (2019). Deep Learning to Assess Long-term Mortality From Chest Radiographs. JAMA Netw open.

[15] Duijm M, van dervan VoortZyp NC, Granton PV, van de Vaart P, Mast ME, Oomen-de Hoop E (2020). Prognostic factors of local control and disease free survival in centrally located non-small cell lung cancer treated with stereotactic body radiation therapy. Acta Oncol.

[16] Kang J, Ning MS, Feng H, Li H, Bahig H, Brooks ED (2020). Predicting 5-Year Progression and Survival Outcomes for Early Stage Non-small Cell Lung Cancer Treated with Stereotactic Ablative Radiation Therapy: Development and Validation of Robust Prognostic Nomograms. Int J Radiat Oncol Biol Phys.

[17] Qiu B, Xiong M, Luo Y, Li Q, Chen N, Chen L (2021). Hypofractionated Intensity Modulated Radiation Therapy With Concurrent Chemotherapy in Locally Advanced Non-Small Cell Lung Cancer: A Phase II Prospective Clinical Trial (GASTO1011). Pract Radiat Oncol.

[18] Kong FM, Ritter T, Quint DJ, Senan S, Gaspar LE, Komaki RU (2011). Consideration of dose limits for organs at risk of thoracic radiotherapy: atlas for lung, proximal bronchial tree, esophagus, spinal cord, ribs, and brachial plexus. Int J Radiat Oncol Biol Phys.

[19] Nioche C, Orlhac F, Boughdad S, Reuzé S, Goya-Outi J, Robert C (2018). LIFEx: A Freeware for Radiomic Feature Calculation in Multimodality Imaging to Accelerate Advances in the Characterization of Tumor Heterogeneity. Cancer Res.

[20] Smola AJ, Scholkopf B (2004). A tutorial on support vector regression. Stat Comput.

[21] Wu MT, Chang JM, Chiang AA, Lu JY, Hsu HK, Hsu WH (1994). Use of quantitative CT to predict postoperative lung function in patients with lung cancer. Radiology.

[22] Lafata KJ, Zhou Z, Liu JG, Hong J, Kelsey CR, Yin FF (2019). An Exploratory Radiomics Approach to Quantifying Pulmonary Function in CT Images. Sci Rep.

[23] Occhipinti M, Paoletti M, Bartholmai BJ, Rajagopalan S, Karwoski RA, Nardi C (2019). Spirometric assessment of emphysema presence and severity as measured by quantitative CT and CT-based radiomics in COPD. Respir Res.

[24] Ganeshan B, Panayiotou E, Burnand K, Dizdarevic S, Miles K (2012). Tumour heterogeneity in non-small cell lung carcinoma assessed by CT texture analysis: a potential marker of survival. Eur Radiol.

[25] Bradley JD, Ieumwananonthachai N, Purdy JA, Wasserman TH, Lockett MA, Graham MV (2002). Gross tumor volume, critical prognostic factor in patients treated with three-dimensional conformal radiation therapy for non-small-cell lung carcinoma. Int J Radiat Oncol Biol Phys.

[26] Lopez Guerra JL, Gomez D, Zhuang Y, Levy LB, Eapen G, Liu H (2012). Change in diffusing capacity after radiation as an objective measure for grading radiation pneumonitis in patients treated for non-small-cell lung cancer. Int J Radiat Oncol Biol Phys.

[27] Park YH, Kim JS (2013). Predictors of radiation pneumonitis and pulmonary function changes after concurrent chemoradiotherapy of non-small cell lung cancer. Radiation Oncol J.

[28] Torre-Bouscoulet L, Muñoz-Montaño WR, Martínez-Briseño D, Lozano-Ruiz FJ, Fernández-Plata R, Beck-Magaña JA (2018). Abnormal pulmonary function tests predict the development of radiation-induced pneumonitis in advanced non-small cell lung Cancer. Respir Res.

[29] Zhou Y, Yan T, Zhou X, Cao P, Luo C, Zhou L (2020). Acute severe radiation pneumonitis among non-small cell lung cancer (NSCLC) patients with moderate pulmonary dysfunction receiving definitive concurrent chemoradiotherapy: Impact of pre-treatment pulmonary function parameters. Strahlentherapie und Onkologie: Organ der Deutschen Rontgengesellschaft [et al].

[30] Dieterich LC, Bikfalvi A (2020). The tumor organismal environment: Role in tumor development and cancer immunotherapy. Sem Cancer Biol.

[31] Khorrami M, Prasanna P, Gupta A, Patil P, Velu PD, Thawani R (2020). Changes in CT Radiomic Features Associated with Lymphocyte Distribution Predict Overall Survival and Response to Immunotherapy in Non-Small Cell Lung Cancer. Cancer Immunol Res.

[32] Dehing-Oberije C, Yu S, De Ruysscher D, Meersschout S, Van Beek K, Lievens Y (2009). Development and external validation of prognostic model for 2-year survival of non-small-cell lung cancer patients treated with chemoradiotherapy. Int J Radiat Oncol Biol Phys.

[33] Antonia SJ, Villegas A, Daniel D, Vicente D, Murakami S, Hui R (2017). Durvalumab after Chemoradiotherapy in Stage III Non-Small-Cell Lung Cancer. N Engl J Med.

[34] Antonia SJ, Villegas A, Daniel D, Vicente D, Murakami S, Hui R (2018). Overall Survival with Durvalumab after Chemoradiotherapy in Stage III NSCLC. N Engl J Med.

[35] Peng L, Wu YL (2018). Immunotherapy in the Asiatic population: any differences from Caucasian population?. J Thorac disease.

[36] Zhao B, Tan Y, Tsai WY, Schwartz LH, Lu L (2014). Exploring Variability in CT Characterization of Tumors: A Preliminary Phantom Study. Translational Oncol.

[37] Lu EL, Li L, Yang L, Schwartz H, Zhao LH (2019). Radiomics for Classification of Lung Cancer Histological Subtypes Based on Nonenhanced Computed Tomography. Acad Radiol.

[38] Cook GJ, Yip C, Siddique M, Goh V, Chicklore S, Roy A (2013). Are pretreatment 18F-FDG PET tumor textural features in non-small cell lung cancer associated with response and survival after chemoradiotherapy? Journal of nuclear medicine: official publication. Soc Nuclear Med.

