# Supplementary material for: CT radiomics-based long-term survival prediction for locally advanced non-small cell lung cancer patients treated with concurrent chemoradiotherapy using features from tumor and tumor organismal environment
Source: Radiat Oncol. 2022 Nov 16;17:184. doi: 10.1186/s13014-022-02136-w (PMC9667605; doi:10.1186/s13014-022-02136-w)
Supplement: Supplementary file 1 — Additional File 1. 4DCT image acquisition [file 13014_2022_2136_MOESM1_ESM.docx]

**Additional File 1** 4DCT image acquisition

| Parameter | Scanner 1 | Scanner 2 | Scanner 3 |
| --- | --- | --- | --- |
|  | SOMATOM Definition AS (Siemens Healthcare, Munich, Germany) | Brilliance^TM^ CT (Philips, Amsterdam, Netherlands) | Brilliance CT Big Bore (Philips, Amsterdam, Netherlands) |
| Detector collimation (mm) | 0.6 | 0.75 | 0.75 |
| Beam pitch | 0.8-1.2 | 1.0 | 1.0 |
| Peak-voltage (kVp) | 140 | 140 | 140 |
| Slice thickness (mm) | 5 | 3-5 | 3-5 |
| Rotation time (s) | 0.33 | 0.75 | 0.5 |
| Tube current (mA) | 20-666 | 250-300 | 240-320 |
| Image matrix | 512×512 | 512 × 512 | 512 × 512 |
