# Supplementary material for: CT radiomics-based long-term survival prediction for locally advanced non-small cell lung cancer patients treated with concurrent chemoradiotherapy using features from tumor and tumor organismal environment
Source: Radiat Oncol. 2022 Nov 16;17:184. doi: 10.1186/s13014-022-02136-w (PMC9667605; doi:10.1186/s13014-022-02136-w)
Supplement: Supplementary file 2 — Additional File 2. Genetic algorithm [file 13014_2022_2136_MOESM2_ESM.docx]

**Genetic algorithm (GA)**

A chromosome in GA was defined as an encoded string of selected features’ identifiers to represent the set of candidate features. For each individual chromosome, a fitness value that represents its suitability was calculated. To calculate this fitness value for one chromosome, the SVM model was trained with features in the chromosome and leave-one-out cross-validation (LOOCV); and the average prediction accuracy of 3-year survival status from LOOCV, representing prediction performance, was employed as the fitness value. Initially, a greedy method with the fitness function as the objective was applied to select one best feature at a time from the remaining feature pool by exhaustive search until the improvement of fitness was marginal. This number of selected features was used to specify the length of the chromosome. The chromosome from the greedy methods together with other n-1 best chromosomes chosen from a large number of randomly generated chromosomes formed the initial population of n individual chromosomes as first generation. Then genetic operations of mutation, crossover and selection were applied sequentially to each individual chromosome to spawn a new generation of n individual chromosomes. As shown in **Figure 2**, the mutation was defined as the operation of an individual chromosome to sequentially replace one feature at a time in chromosome with the best feature in unselected feature pool, while keeping all other features fixed, until loop over all the features. The crossover was defined as the operation of two individual chromosomes to exchange part of the chromosome with each other. The pairing of chromosomes for crossover was random. The selection was defined as the operation of selecting best n/2 chromosomes from the last generation and the current generation in preparation, and selecting the n/2 chromosomes from the remaining chromosomes in a stochastic fusion with each chromosome to be selected by a normalized probability proportional to its fitness. Generations of chromosomes were spawned until there was no discernible improvement in the fitness of all the chromosomes or the number of generations reached a predefined limit.
