# Supplementary material for: CT radiomics-based long-term survival prediction for locally advanced non-small cell lung cancer patients treated with concurrent chemoradiotherapy using features from tumor and tumor organismal environment
Source: Radiat Oncol. 2022 Nov 16;17:184. doi: 10.1186/s13014-022-02136-w (PMC9667605; doi:10.1186/s13014-022-02136-w)
Supplement: Supplementary file 3 — Additional File 3 Patient characteristics [file 13014_2022_2136_MOESM3_ESM.docx]

**Additional File 3** Patient characteristics

| **Characteristic** | **Training Cohort** | **Validation Cohort** | ***P*-value** |
| --- | --- | --- | --- |
|  | ***N*=200 (%)** | ***N*=98 (%)** |  |
| Age (years) |  |  | *0.46* |
| Median (range) | 58 (28~81) | 61 (31~77) |  |
| Sex |  |  | *0.64* |
| Male | 160 (80.0) | 81 (82.7) |  |
| Female | 40 (20.0) | 17 (17.3) |  |
| ECOG PS |  |  | *0.70* |
| 0 | 49 (24.5) | 22 (22.4) |  |
| 1 | 139 (69.5) | 72 (76.5) |  |
| 2 | 12 (6.0) | 4 (4.1) |  |
| Pathology |  |  | *0.038* |
| Squamous cell carcinoma | 93 (46.5) | 61 (62.2) |  |
| Adenocarcinoma | 85 (42.5) | 31 (31.6) |  |
| Lymphoepithelioma-like carcinoma | 13 (6.5) | 3 (3.1) |  |
| Adeno-squamous cell carcinoma | 2 (1.0) | 0 (0) |  |
| Adenoid cystic carcinoma | 2 (1.0) | 0 (0) |  |
| Large cell carcinoma | 0 (0) | 2 (2.0) |  |
| NSCLC, NOS | 5 (2.5) | 1 (1.0) |  |
| Lung comorbidity |  |  | *0.20* |
| Yes | 70 (35.0) | 42 (42.9) |  |
| No | 130 (65.0) | 56 (57.1) |  |
| Smoking index |  |  | *0.46* |
| ≥400 | 110 (55.0) | 59 (60.2) |  |
| <400 | 90 (45.0) | 39 (39.8) |  |
| T stage |  |  | *0.91* |
| Tx | 1 (0.5) | 1(1.0) |  |
| T1 | 19 (9.5) | 7 (7.1) |  |
| T2 | 77 (38.5) | 36 (36.7) |  |
| T3 | 45 (22.5) | 25 (25.5) |  |
| T4 | 58 (29.0) | 29 (29.6) |  |
| N stage |  |  | *0.97* |
| N0 | 4 (2.0) | 2 (2.0) |  |
| N1 | 14 (7.0) | 7 (7.1) |  |
| N2 | 80 (40.0) | 42 (42.9) |  |
| N3 | 102 (51.0) | 47 (48.0) |  |
| Stage |  |  | *0.92* |
| IIIA | 55 (27.5) | 27 (27.6) |  |
| IIIB | 104 (52.0) | 49 (50.0) |  |
| IIIC | 41 (20.5) | 22 (22.4) |  |
| Radiation dose (Gy) |  |  | *0.62* |
| Median | 65.0 | 65.0 |  |
| Range | 60.0~70.4 | 60.0~68.1 |  |
| GTV (cm^3^) |  |  | *0.18* |
| Median | 98.6 | 117.6 |  |
| Range | 9.1~664.3 | 17.7~567.7 |  |
| Total volume of lungs (cm^3^) |  |  | *0.54* |
| Median | 3191.1 | 3268.7 |  |
| Range | 1487.0~5605.1 | 1561.6~5803.0 |  |
| Pulmonary function test * |  |  |  |
| FEV1/FVC% |  |  | *0.88* |
| Median (range) | 90.9 (48.0-118.4) | 90.5 (49.9-124.5) |  |
| DLCO% |  |  | *0.66* |
| Median (range) | 78.3 (47.5-110.4) | 80.6 (35.7-113.2) |  |
| Blood gas analysis ^#^ |  |  |  |
| pO2 (mmHg) |  |  | *0.32* |
| Median (range) | 87.5 (63.2-150.8) | 86.0 (56.6-106.4) |  |
| AaDO2 (mmHg) |  |  | *0.77* |
| Median (range) | 17.6 (0.4-116.3) | 18.0 (3.2-45.7) |  |
| SaO2 (%) |  |  | *0.39* |
| Median (range) | 96.8 (91.9-99.4) | 96.6 (89.2-98.4) |  |
| Lymphocyte counts (cells/mm^3^) |  |  |  |
| Before CCRT |  |  | *0.39* |
| Median (range) | 1800 (700-3600) | 1650 (500-3350) |  |
| The Lowest during CCRT |  |  | *0.54* |
| Median (range) | 400 (10-1210) | 300 (80-1400) |  |
| 4~6 weeks post CCRT ^&^ |  |  | *0.12* |
| Median (range) | 1100 (200-3430) | 900 (200-2600) |  |

Abbreviations: ECOG PS, Eastern Cooperative Oncology Group performance status; NSCLC, non-small cell lung cancer; NOS, not otherwise specified; GTV, gross tumor volume; CCRT, concurrent chemoradiotherapy.

* The pretreatment pulmonary function test information was available for 205 patients.

^#^ The pretreatment blood gas analysis information was available for 220 patients.

^&^ Twenty-two patients had no record of lymphocyte counts at 4~6 weeks post CCRT.
