# Supplementary material for: CT radiomics-based long-term survival prediction for locally advanced non-small cell lung cancer patients treated with concurrent chemoradiotherapy using features from tumor and tumor organismal environment
Source: Radiat Oncol. 2022 Nov 16;17:184. doi: 10.1186/s13014-022-02136-w (PMC9667605; doi:10.1186/s13014-022-02136-w)
Supplement: Supplementary file 4 — Additional File 4. Tumor response after CCRT in the low-risk and high-risk group [file 13014_2022_2136_MOESM4_ESM.docx]

**Additional File 4** Tumor response after CCRT in the low-risk and high-risk group

|  | Low-risk group  (n=38) | High-risk group  (n=60) | *P*-value |
| --- | --- | --- | --- |
| Response, n (%) |  |  |  |
| CR | 7 (18.4) | 0 (0) |  |
| PR | 25 (65.8) | 40 (66.7) |  |
| SD | 4 (10.5) | 14 (23.3) |  |
| PD | 2 (5.3) | 6 (10.0) |  |
| ORR (%) | 84.2 | 66.7 | 0.003 |

Abbreviations: CCRT, concurrent chemoradiotherapy; CR, complete remission; PR, partial remission; SD, stable disease; PD, progressive disease; ORR, objective response rate.
