# Supplementary figures and images for: CT radiomics-based long-term survival prediction for locally advanced non-small cell lung cancer patients treated with concurrent chemoradiotherapy using features from tumor and tumor organismal environment
Source: Radiat Oncol. 2022 Nov 16;17:184. doi: 10.1186/s13014-022-02136-w (PMC9667605; doi:10.1186/s13014-022-02136-w)

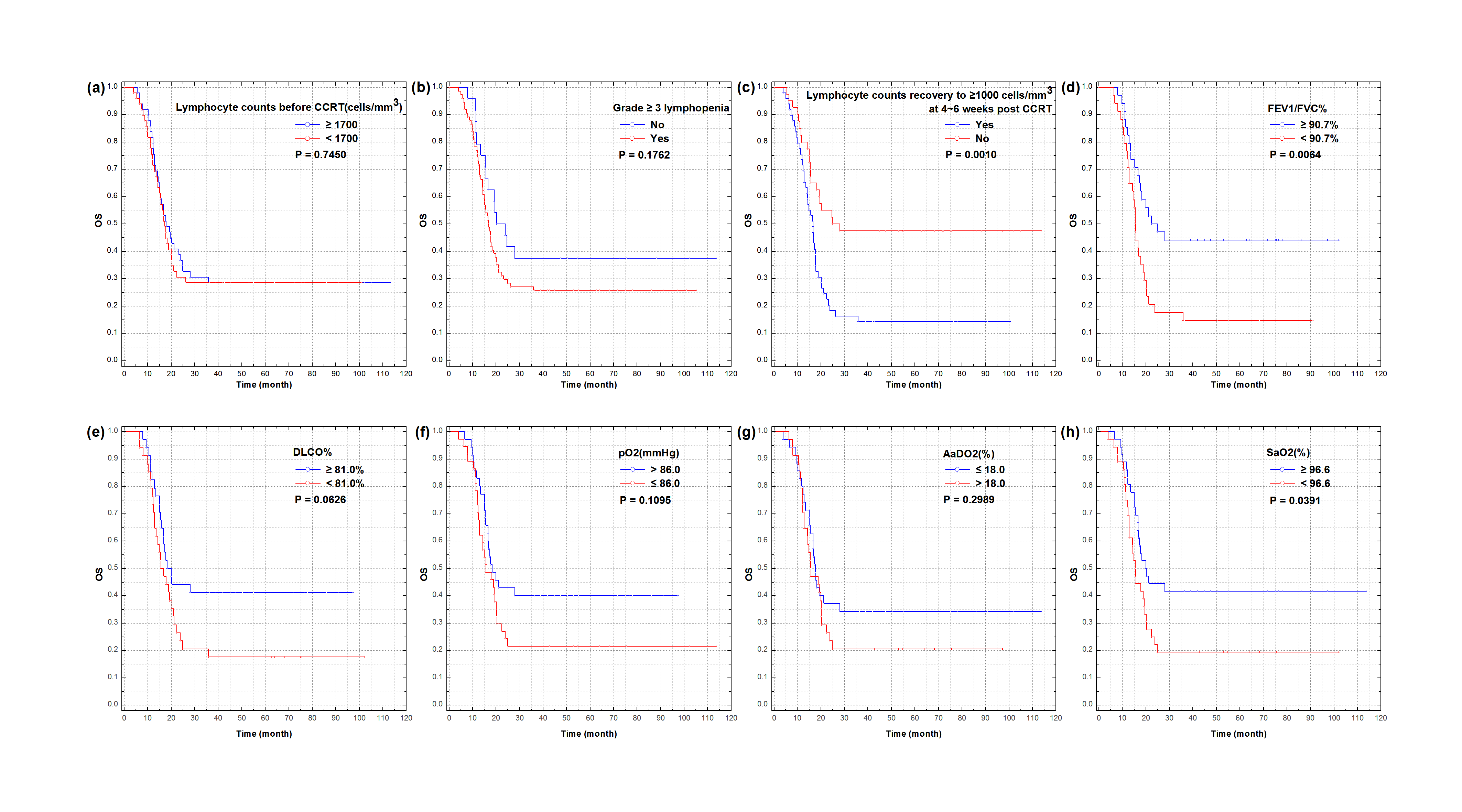

Supplement: Supplementary file 5 — Additional File 5. [file 13014_2022_2136_MOESM5_ESM.png]

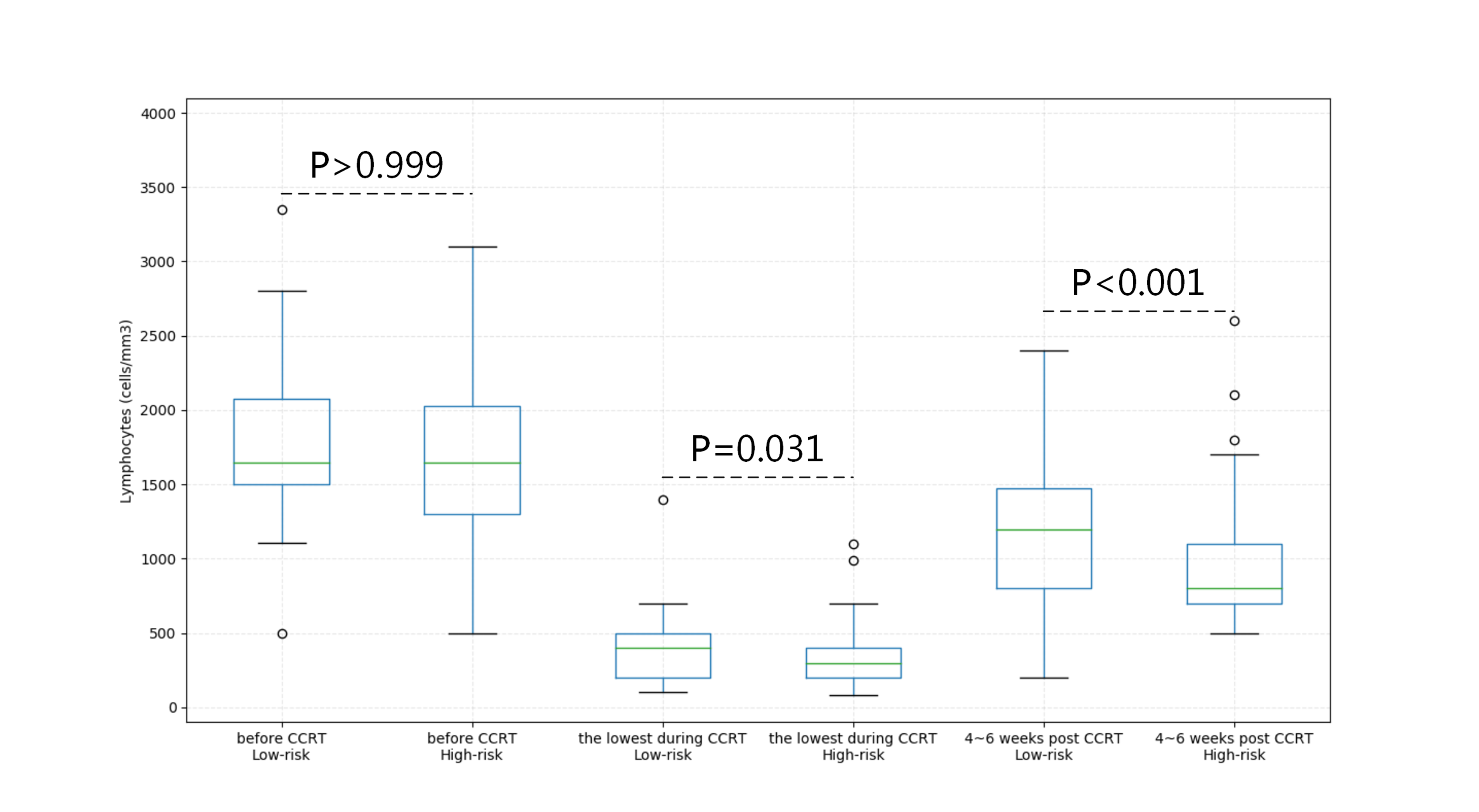

Supplement: Supplementary file 6 — Additional File 6. [file 13014_2022_2136_MOESM6_ESM.png]
